# Mapping the landscape and research trend of imaging diagnosis in lymphoma: a bibliometric analysis from 1976 to 2024

**DOI:** 10.3389/fmed.2025.1516817

**Published:** 2025-01-29

**Authors:** Yi Ma

**Affiliations:** Department of Radiology, Beijing Puren Hospital, Beijing, China

**Keywords:** imaging diagnosis, lymphoma, bibliometric analysis, MR, CT

## Abstract

**Background:**

Over the past five decades, extensive research has been conducted on lymphoma imaging diagnostics; however, no bibliometric analysis has been performed in this area. Therefore, we undertook a bibliometric analysis to clarify the progress and current state of research in this field.

**Methods:**

We conducted a search of the Web of Science Core Collection database for articles related to imaging diagnosis and lymphoma, focusing exclusively on English-language publications up to June 20, 2024. We analyzed and visualized various aspects, including publication trends, journals, co-authorship networks, countries, institutions, and keywords. To examine research trends in this field, we utilized bibliometric analysis tools such as VOSviewer, CiteSpace, and R4.3.3.

**Results:**

From 1976 to 2024, a total of 10,410 publications were produced on this topic, with 2021 marking the peak in publication numbers. The most significant contributions in this research area were found in the fields of *Radiology*, *Nuclear Medicine & Medical Imaging*, *Oncology*, and *Hematology*. The United States, China, and Japan were the leading contributors. Zucca Emanuele ranked first among authors, followed closely by Meignan Michel. In terms of institutions, Assistance Publique Hôpitaux de Paris was the most prominent. The most frequently used keywords included positron emission tomography, computed tomography, and non-Hodgkin’s lymphoma.

**Conclusion:**

This study presented a bibliometric analysis of lymphoma imaging diagnosis, highlight showcasing research trends, influential significant studies, and collaborative networks. The analysis identified key contributions to the field and provide insights for future research directions.

## Introduction

Lymphomas constitute a group of hematologic malignancies that primarily manifest in the lymph nodes and represent a substantial threat to global health, given the reported incidence of over 627,000 new cases and 283,000 fatalities worldwide in the year 2020 alone (1). Notably, the incidence of lymphoma, particularly non-Hodgkin lymphoma (NHL), has increased by approximately 3–4% over the past few decades (2). Hodgkin lymphoma (HL) also contributes significantly to the global cancer burden, with an estimated 83,000 new cases annually (3). Both HL and NHL present severe health risks if not promptly diagnosed and treated (4). Effective diagnostic tools and therapeutic interventions are urgently required. Current treatment strategies include chemotherapy, radiation therapy, targeted therapies, and immunotherapy, all of which need to be tailored to the specific type and stage of lymphoma (5). However, the success of these treatments depends heavily on early and accurate diagnosis, which has been greatly improved by advances in imaging technology.

Imaging modalities such as computed tomography (CT), magnetic resonance imaging (MRI), and positron emission tomography (PET) have become integral in the diagnosis, staging, and monitoring of lymphoma. PET/CT, in particular, has revolutionized the management of lymphoma by providing more accurate staging and allowing for the detection of residual active disease (6, 7). Despite significant advancements in these technologies, the research progress in imaging diagnostics for lymphoma remains unclear, particularly in terms of how these technologies are applied across different lymphoma subtypes, their impact on clinical decision-making, and the consistency of their application in clinical practice (8). While imaging techniques have demonstrated substantial value, challenges still persist in understanding their evolving roles, diagnostic accuracy, and integration into treatment regimens (9).

Conducting a bibliometric analysis in this field is of paramount importance due to the increasing complexity of imaging diagnostics, particularly with the integration of advanced domains such as molecular imaging and artificial intelligence (10, 11). This necessitates a systematic approach to track and evaluate the progress in imaging diagnostics for lymphoma. Bibliometric analysis serves as a powerful tool to map research trends, evaluate the influence of various studies, and identify emerging areas for future research (12). Although existing research focuses on various aspects of lymphoma treatment and diagnosis, including chemotherapy and immunotherapy, bibliometric analyses in the field of lymphoma have primarily addressed the application of traditional Chinese medicine in treatment and diffuse large B-cell lymphoma (13, 14). A significant gap exists in the area of imaging diagnostics for lymphoma. Understanding this gap is crucial, as the application of imaging technologies directly influences clinical outcomes, treatment strategies, and patient survival rates (15). Considering the critical role of imaging diagnosis of lymphoma and the necessity for an in-depth understanding of current research trends, this study aims to conduct a bibliometric analysis of the literature on imaging diagnosis of lymphoma, identifying research trends, key focus areas, and significant collaborations to guide future studies and clinical practices.

## Materials and methods

### Search strategies and data collection

We conducted the literature search on the Web of Science Core Collection (WoSCC). The search formula was as follows: (TS = (lymphoma)) AND TS = (“imaging diagnosis” OR “Computed Tomography” OR “CT” OR “PET-CT” OR “Positron Emission Tomography-Computed Tomography” OR “MRI” OR “Magnetic Resonance Imaging” OR “Nuclear Magnetic Resonance Imaging” OR “NMRI” OR “abdominal ultrasonography”). To minimize potential bias from database updates, the search was performed on June 20, 2024. Data collected included the number of publications and citations, titles, author details, institutions, countries/regions, keywords, and journals, all formatted as text. The study was restricted to articles published in English. After careful screening, a total of 10,410 eligible publications were included in the present analysis.

### Statistical analysis

For the visualization analysis, we employed three powerful bibliometric analysis tools to comprehensively analyze the academic data. These tools were VOSviewer (version 1.6.20), CiteSpace (version 6.3.R1), and the R (version 4.3.3). We used the Bibliometrix package in R version 4.3.3 to extract and analyze basic information about publications (16). VOSviewer is a versatile software tool that plays a critical role in mapping institutional collaboration, author collaboration, co-authorship, citation, and co-citation (17). It enabled us to explore and visualize intricate academic networks, uncovering valuable insights into the relationships among authors, institutions, and research outputs. To identify emerging trends and research hotspots, we performed keyword co-occurrence analysis in VOSviewer and keyword burst detection using CiteSpace.

For the analysis of keyword burst detection using CiteSpace version 6.1.R3, we applied the following parameters: the time slicing was set from January 1994 to June 2024, with the node type defined as keywords. The threshold included the top 5 keywords for each time slice. For pruning, we applied the pathfinder algorithm and the pruning merged network technique. This allowed us to generate visualized co-occurrence networks where node size indicated the number of publications, line thickness represented link strength, and node color corresponded to different clusters or times.

R-bibliometrix is an important R-tool for comprehensive bibliometric analysis (18). Its functions encompass creating bibliographic coupling networks, co-citation networks, co-authorship networks, and co-occurrence networks. With a streamlined process for data import, transformation, analysis, and visualization, Bibliometrix effectively meets the demands of bibliometric studies.

To identify and calculate bibliometric metrics, we used Microsoft Excel 16. These indicators cover key aspects of the publications, including the annual number of publications, citation frequency, average citation frequency, journal names, journal impact factors (IF), countries/regions of publication, publishing institutions, and authors. Based on the IF of journals in their respective academic fields, journal citation reports (JCR) divide them into four quartiles: Q1 (top 25%), Q2 (25–50%), Q3 (50–75%), and Q4 (bottom 25%). The latest data for 2023 was used for both IF and JCR quantiles in this study. On this basis, we employed H-index, M-index, and G-index to quantify the academic impact of individuals and journals, respectively. The H-index is the most widely used bibliometric metric and is defined as the number of papers *h* cited at least *h* times (19). The G-index is introduced as an improvement of the H-index to measure the global citation performance of a set of articles (20), whereas the M-index is the average number of citations received by the papers that make up the H-index (21). In this study, the H-index of each author was obtained from WoSCC.

## Results

### Publications landscape on imaging diagnostics in lymphoma

**FIGURE 1:**
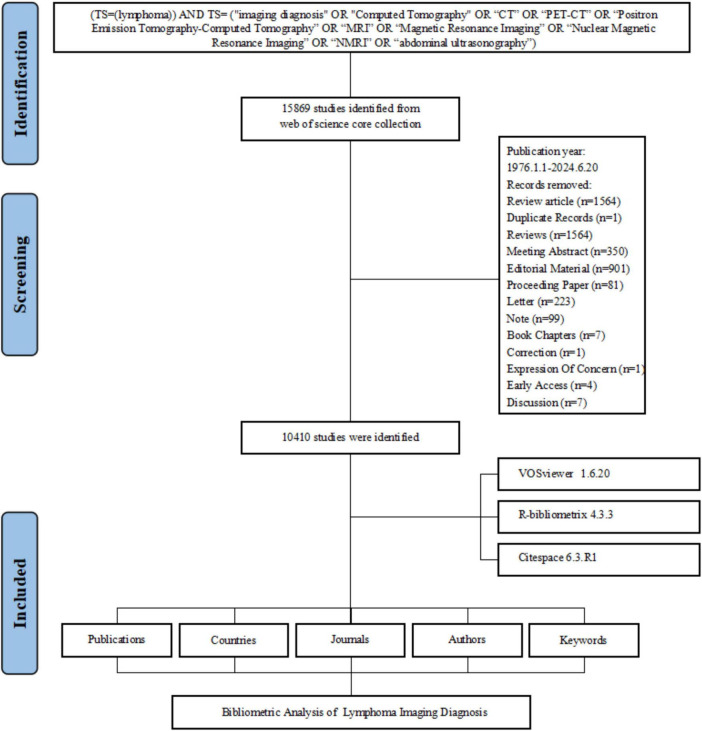
Flowchart of the literature screening process.

**FIGURE 2:**
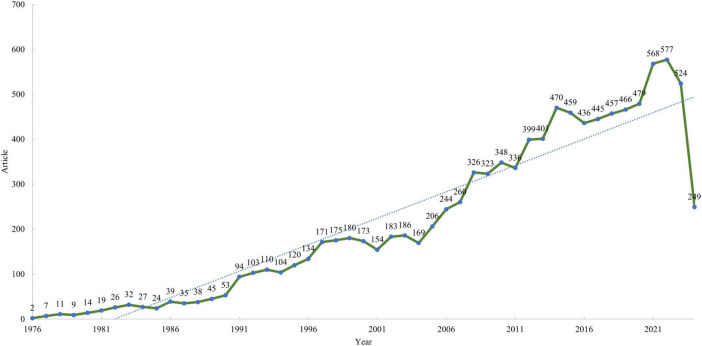
Annual publication numbers from 1976 to 2024.

The flowchart of data screening is shown in Figure 1. This bibliometric analysis, encompassing research on imaging diagnostics of lymphoma from 1976 to 2024, identified 10,410 documents from 1,509 sources. The annual growth rate of publications was 10.57%, with contributions from 55,515 authors and an average of 7.24 co-authors per document. Notably, 11.82% of the studies involved international collaborations. The dataset included 13,262 unique author keywords and 155,005 references, with each document averaging 24.53 citations. The average age of documents was 12.9 years, reflecting a mix of foundational and contemporary research. The annual publication has grown in the area for nearly 50 years, achieving its maximum peak in 2022, with 577 documents in overall imaging diagnosis of lymphoma research. The overall trend shows a smooth upward trajectory, with a significant increase beginning in 2008 (Figure 2).

The most frequently cited publications are as follows. The most-cited publication, titled “Recommendations for initial evaluation, staging, and response assessment of Hodgkin and non-Hodgkin lymphoma: the Lugano classification” was published in the *Journal of clinical oncology* (IF = 42.1) in 2014, and has received 3,309 citations (6). The second cited article is “Report of an international workshop to standardize response criteria for non-Hodgkin’s lymphomas. NCI Sponsored International Working Group” published in the *Journal of clinical oncology* (IF = 42.1) in 1999, with a total of 2,902 citations (22). The third cited article is “From RECIST to PERCIST: Evolving Considerations for PET response criteria in solid tumors” published in *Journal of nuclear medicine* (IF = 9.1) in 2009, with a total of 2,757 citations (23).

### Journal insights and trends in lymphoma research

The general characteristics of the 20 most productive journals in imaging diagnostics of lymphoma between 1976 and 2024 are summarized in Supplementary Table S1. These journals collectively published 2,294 articles, accounting for 22.04% of all retrieved publications. High-impact journals in the fields of Radiology, Nuclear Medicine & Medical Imaging, Oncology, and Hematology significantly shaped this area of research. The most prolific journals ( > 100 articles) were *Radiology* (IF: 12.1), *Journal of Clinical Oncology* (IF: 42.1), *Journal of Nuclear Medicine* (IF: 9.1), *American Journal of Roentgenology* (IF: 4.7), *European Journal of Nuclear Medicine and Molecular Imaging* (IF: 8.6), *Journal of Computer Assisted Tomography* (IF: 1.0), *European Journal of Radiology* (IF: 3.2), *European Radiology* (IF: 4.7), and *Leukemia & Lymphoma* (IF: 2.2). Together, these journals published approximately 14.7% (*n* = 1,533) of all articles, highlighting their pivotal role in disseminating research on imaging diagnostics in lymphoma.

### Country insights and trends in lymphoma research

**FIGURE 3:**
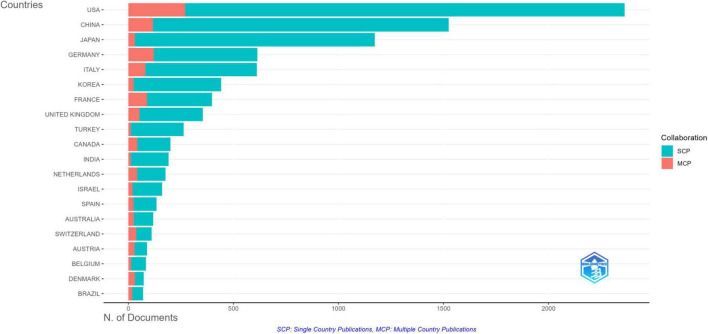
Top 20 prolific countries of corresponding authors in publishing papers on imaging diagnosis of lymphoma (1976–2024).

**FIGURE 4:**
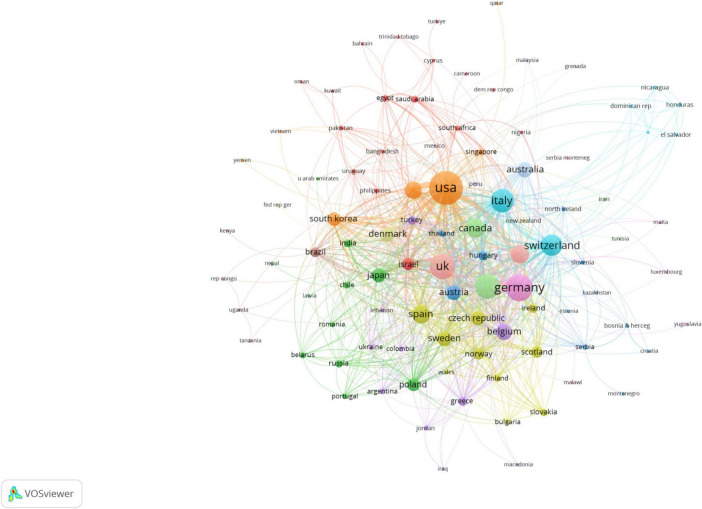
Visualization map depicting the collaboration among different countries.

A total of 2,306 countries published articles in this field. The top 20 productive countries generated 9,149 articles, accounting for 92.2% of the papers worldwide. The United States was the most productive country with the highest articles (*n* = 2,362), followed by China (*n* = 1,525) and Japan (*n* = 1,173). In terms of citations, the United States amassed the highest number, with 91,816 citations. Germany followed with 18,737 citations, Japan with 17,491 citations, and China with 15,537 citations (Supplementary Table S2). Research results and patterns of collaboration across countries showed that Denmark (MCP ratio = 42.5%, *n* = 73), Switzerland (MCP ratio = 33.3%, *n* = 111), Austria (MCP ratio = 32.6%, *n* = 89), and Brazil (MCP ratio = 27.1%, *n* = 70) were the countries collaborating more actively (Figure 3). In addition, 103 countries with a minimum of 1 document in co-authorship were analyzed with VOSviewer (Figure 4 and Supplementary Table S3). Among them, the United States has the highest number of collaborations with other countries (1,155), followed by the United Kingdom (727) and Italy (643) regarding total link strength.

### Authors insights and trends in lymphoma research

**FIGURE 5:**
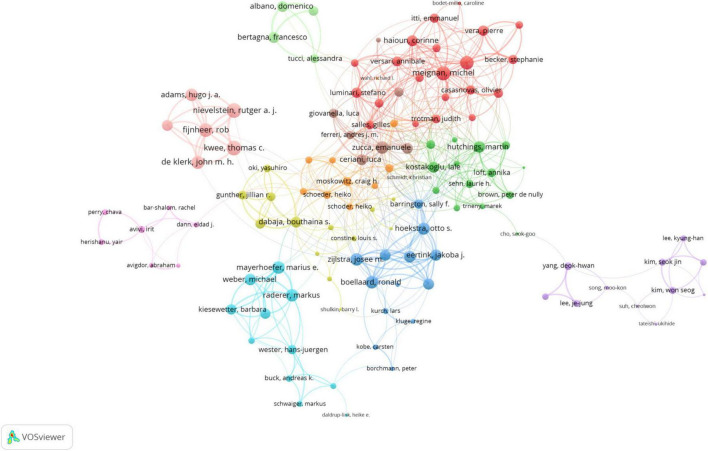
Visualization map depicting the collaboration among different authors.

A total of 55,515 authors contributed to this field’s publications. The top 20 most cited authors are listed in Supplementary Table S4. Zucca Emanuele was the most cited author (cited 5,195 times, h-index = 15), followed by Meignan Michel (cited 3,656 times, h-index = 21) and Wahl Richard L. (cited 3,499 times, h-index = 14). In addition, based on the duration of their academic careers, the m-index showed that these authors (Albano Domenico = 2.11; Mayerhoefer Marius E. = 1.25; Meignan Michel = 1.18; Tilly Herve = 1.06; Hutchings Martin = 1.0; Kostakoglu Lale = 1.0; Luminari Stefano = 1.0) experienced significant advancements in their scientific output. The co-authorship network of research findings on imaging diagnosis of lymphoma is visualized in Figure 5, with the research results broadly categorized into 11 groups. Among the 163 authors involved in international collaborations with a minimum of 11 articles, Kwee Thomas C. has the highest number of collaborations with other authors (total link strength = 107), followed by Nievelstein Rutger A. J. (total link strength = 105) and Meignan Michel (total link strength = 98).

### Institutions insights and trends in lymphoma research

**FIGURE 6:**
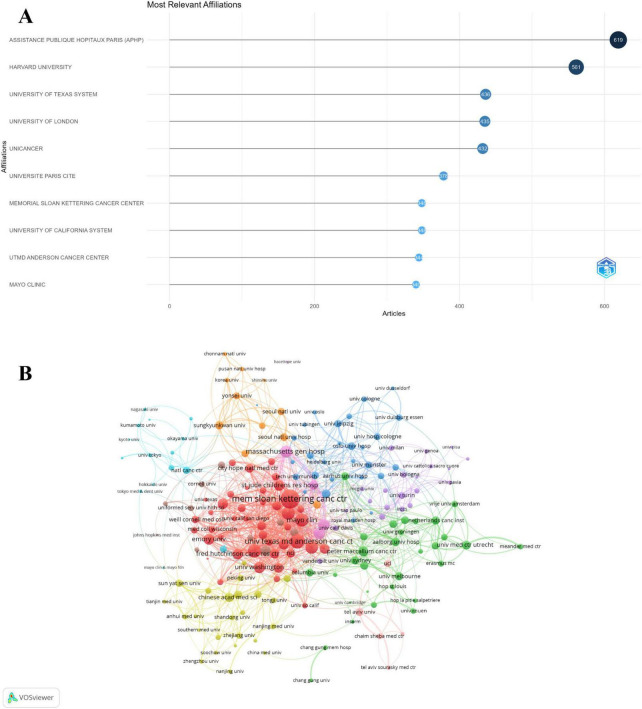
Analysis of institutions. **(A)** Top ten institutions by article count and rank. **(B)** Visualization networks of institution collaborations.

A total of 35,540 institutions were involved in this field. The top 10 institutions with the highest in research are displayed in Figure 6A. The Assistance Publique Hopitaux De Paris (APHP) in Paris (*n* = 619) was the leading institution, followed by Harvard University in the United States (*n* = 561) and the University of Texas System (*n* = 436). In addition, our study revealed the cooperative relationship among 195 institutions that published a minimum of 21 documents. It divides institutions into seven clusters (Figure 6B). In the top 3 big cooperation groups, Memorial Sloan Kettering Cancer Center has the highest number of collaborations with other countries (362), followed by the University of Texas MD Anderson Cancer Center (219) and Mayo Clinic (181).

### Keywords insights and trends in lymphoma research

**FIGURE 7:**
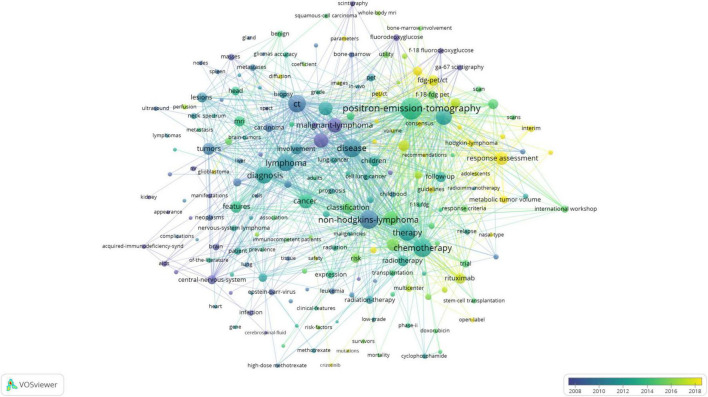
Visual analysis of keyword co-occurrence network analysis.

Keywords encapsulate research topics and core content. Conducting a keyword co-occurrence analysis enables us to understand the distribution and evolution of research hotspots. The keywords were filtered from the existing data, and a total of 197 words were obtained (Figure 7). The top five keywords with the most frequent occurrence were “PET,” “lymphoma,” “disease,” “CT,” and “NHL,” the top five keywords reflecting outcomes were “lymphoma,” “NHL,” “cancer,” “B-cell lymphoma,” and “malignant lymphoma,” and the top five keywords reflecting diagnostic tools were “CT,” “fluorodeoxyglucose (FDG) PET,” “MRI,” and “FDG PET-CT.” In addition, the color of the nodes, from purple, blue, green to yellow, corresponds to the earliest to most recent keywords that were used in the publications (24), reflecting which keywords have become popular in recent years and indicating the trend of future hotspots (25). The research focus in the field has undergone significant changes over time. Between 2008 and 2012, key topics included “acquired immunodeficiency syndrome,” “malignant lymphoma,” “FDG,” “CT,” “lymphoma,” and “NHL.” From 2012 to 2016, the focus gradually shifted toward “PET,” “diagnosis,” “MRI,” “cancer,” “chemotherapy,” etc. In the subsequent period from 2016 to 2018, emerging research areas included “FDG,” “HL,” “metabolic tumor volume,” and “rituximab,” reflecting the current hotspots and frontiers of scientific research.

### Identifying emerging themes through keyword bursts

**FIGURE 8:**
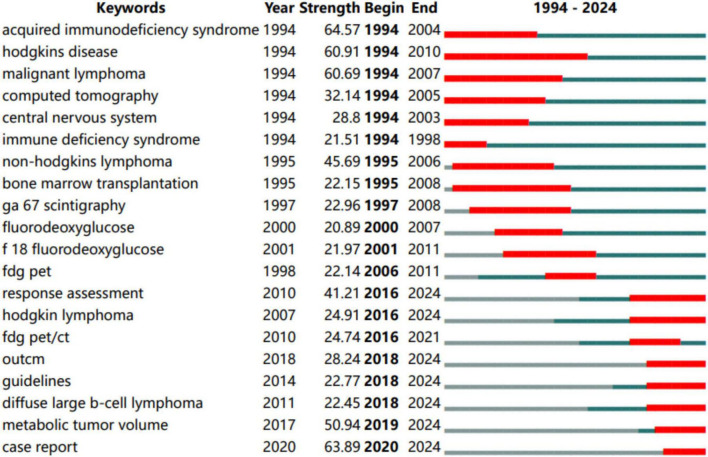
Top 20 keywords with the strongest citation bursts (CiteSpace).

The burst analysis of keywords in a slice from 1994 to 2024 was performed to reveal the evolution trend. The blue lines stand for the time span. The red lines represent the burst period. The strength of the top 20 keywords with the strongest bursts varied from 20.89 to 64.57 (Figure 8). The term acquired immunodeficiency syndrome owned the highest burst strength from 1994 to 2004. Our study reveals that the term “acquired immunodeficiency syndrome” has the highest burst intensity, followed by “case reports,” “Hodgkin disease,” “metabolic tumor volume,” and “response assessment.” The term “Hodgkins disease” had the longest time span of outbreaks, followed by “malignant lymphoma,” and “bone marrow transplantation.” It is worth noting that in recent years there has been a stronger keyword “FDG PET-CT” fever about the means of diagnostic imaging, higher than “diffuse large B-cell lymphoma” and lasted for 5 years. In summary, the keyword analysis provides a research trajectory of lymphoma imaging diagnosis, including major research areas, current research concerns, and future research trends.

## Discussion

Bibliometric analysis has evolved into a paramount tool for exploring nuanced research trends within a specific domain. Notably, however, no bibliometric analysis has hitherto been conducted on the imaging diagnosis of lymphoma, with scant attention devoted to predicting research hotspots in this realm. Our research conducted a bibliometric analysis of literature regarding the imaging diagnosis in lymphoma over the last 50 years. Generated maps and tables to help decipher the research status uncovering hotspots and emerging trends, as well as to explor the intellectual structure of this domain intuitively. In general, the past five decades have seen a gradual increase in the numbers of publications on this topic, and reaching a peak in 2022.

### General information

High-impact factor journals dominate the field, such as *Radiology* (IF: 12.1) and *Journal of Clinical Oncology* (IF: 42.1). The *Journal of Clinical Oncology* focuses on clinical oncology research and provides a platform for showcasing new technologies and methods in oncologic imaging diagnosis. The research published in this journal often initiates new rounds of clinical trials and technology applications (26). Besides its high impact factor, the journal is also popular among scholars because its published research findings are more likely to receive recognition and citations from peers (27).

As evidenced by our findings, the United States was the leading contributor in the lymphoma imaging diagnosis field with 2,362 publications, followed by China, and Japan, which may be related to open and reasonable academic policies, national conditions, and economic growth, resulting in better financial support for research. For example, the U.S. Cancer to the Moon Initiative has promoted research on early diagnosis and treatment of cancer through large-scale funding and policy support (28). Similarly, China’s Healthy China 2030 strategy has led to breakthroughs in research on major diseases such as cancer (29). In the future, these countries can expand international research alliances to further advance the application of artificial intelligence and imaging diagnostic technologies in lymphoma management (30).

Among these countries, Harvard University and Texas System University in the United States and APHP in France not only have abundant resources but also actively engage in international collaborations globally. APHP, as one of the largest hospital networks in Europe, also has abundant resources of collaborative organizations and actively participates in a number of important research projects in the field of cancer diagnostic imaging (31). In turn, author Meignan Michel has relied on APHP’s rich resources to demonstrate a strong presence in the academic community, excelling in the number of publications, citations, and collaborative networks (32).

Regarding citation count, Zucca Emanuele has made significant contributions to the field of lymphoma imaging, particularly in establishing the role of PET-CT in staging and response assessment. His team participated in the development of the consensus of the International Conference on Malignant Lymphoma (15). Meignan Michel has advocated for the need for standardized criteria in clinical practice, emphasizing the advancement of PET imaging for lymphoma diagnosis (33). In the future, leading experts can strengthen diagnostic and therapeutic standardization by spearheading international working groups and conducting multicenter clinical trials. For instance, the International Primary CNS Lymphoma Collaborative Group (IPCG), through multinational research efforts, has significantly contributed to the standardization of MRI and PET imaging protocols for clinical evaluation (34).

### Key findings

The analysis highlights major trends in imaging diagnosis of lymphoma. Frequently occurring keywords, including “PET,” “CT,” and “NHL,” reflects the pivotal role of imaging techniques in lymphoma diagnosis (14). From 2008 to 2012, keywords like “acquired immune deficiency syndrome” and “malignant lymphoma” indicated a focus on utilizing imaging for immunosuppression-related malignancies, closely related to the AIDS outbreak in the 1990s (35).

Looking at the evolution of keywords from 2016 to 2018, new areas of interest such as “metabolic tumor volume (MTV),” “HL,” and “rituximab” surfaced. These reflect a shift toward precision medicine, emphasizing quantitative imaging biomarkers and targeted therapies. For example, studies have shown that high MTV is linked to worse outcomes in diffuse large B-cell lymphoma, with higher MTV associated with lower survival rates (36). Additionally, combining rituximab with MTV assessment has enhanced treatment stratification, highlighting its growing role in guiding therapy (37).

Over time, research has gradually shifted toward more precise imaging diagnostic tools, such as “PET,” “MRI,” and the “integration.” Since the burst in 2016, FDG PET-CT has emerged as a pivotal tool in the diagnosis and management of lymphoma, offering superior sensitivity and specificity in detecting metabolically active disease. Its inclusion in clinical guidelines underscores its critical role in modern lymphoma care (6). FDG PET-CT’s ability to provide detailed metabolic information, which complements anatomical imaging, has made it indispensable for staging, treatment response assessment, and detecting residual or recurrent disease. Recent studies have further highlighted its utility in distinguishing viable tumor tissue from post-treatment changes, thus guiding therapeutic decisions and improving patient outcomes (38). Moreover, FDG PET-CT has been instrumental in evaluating extranodal involvement, which is crucial in certain lymphoma subtypes where conventional imaging might fall short (39). In addition, the integration of artificial intelligence (AI) with FDG PET-CT has introduced a transformative dimension to imaging diagnosis. AI algorithms are increasingly applied to interpret complex imaging data, enhancing diagnostic precision while reducing human error. For example, AI-driven radiomics can extract quantitative features from PET images, providing valuable prognostic insights and enabling personalized treatment planning. Furthermore, AI has the potential to optimize imaging protocols, reducing scan times and improving image quality, particularly for challenging cases involving small lesions or low metabolic activity (40). This synergy between AI and FDG PET-CT not only improves diagnostic accuracy but also paves the way for a more tailored approach to lymphoma management, which is likely to remain a key research focus in the coming years (41).

While FDG PET-CT is often the modality of choice for whole-body imaging, MRI serves a complementary role, particularly in assessing central nervous system involvement and bone marrow infiltration (42). The superior soft-tissue contrast of MRI makes it an excellent tool for evaluating areas that are challenging to assess with FDG PET-CT alone. Combined PET/MRI approaches are increasingly being recognized for their ability to provide comprehensive diagnostic information, integrating metabolic data from PET with the high-resolution anatomical detail provided by MRI (43).

The analysis of keywords over time reveals significant shifts in research focus, with FDG PET-CT emerging as a key area of interest in recent years. The burst analysis highlights FDG PET-CT’s sustained impact on the field, suggesting that it will remain a central focus of research, especially as new radiotracers and imaging protocols are developed to enhance its diagnostic and prognostic capabilities (44). Additionally, the increasing use of MRI, particularly in combination with PET, points to a trend toward more integrated and comprehensive imaging approaches in lymphoma diagnosis (45).

### Implications for future research

The findings from this bibliometric analysis highlight the transformative potential of integrating advanced imaging modalities with AI in the diagnosis and management of lymphoma. FDG PET-CT remains pivotal in providing metabolic and anatomical insights, and its synergy with MRI enhances diagnostic accuracy and treatment planning. Future research is expected to emphasize the role of AI-driven systems, which have shown superior performance in automating diagnostic tasks and prognostic evaluations. For instance, AI-based radiomics derived from PET/CT imaging can identify subtle tumor characteristics that surpass human visual interpretation, enhancing risk stratification and personalized treatment strategies (46). Additionally, novel AI algorithms utilizing MRI have demonstrated high accuracy in distinguishing lymphoma subtypes and predicting survival outcomes, facilitating tailored therapeutic interventions (47). The continued development of automated segmentation tools for PET-based biomarkers such as total metabolic tumor volume (TMTV) holds promise for improving staging and treatment monitoring (48). Furthermore, incorporating AI into hybrid imaging systems like PET/MRI is expected to enhance multimodal data integration, paving the way for breakthroughs in precision oncology. These advancements underscore the critical need for interdisciplinary collaboration to fully harness AI’s capabilities in lymphoma care.

### Limitations

This study has several potential limitations. First, relying on citation counts may not fully reflect an article’s clinical impact. Second, excluding non-English publications could narrow the scope of the analysis. Third, the keywords used in the analysis are self-defined by the authors, which may affect the accuracy of the keyword co-occurrence results. Future research should aim to standardize keyword usage across scientific studies to improve consistency and comparability.

## Conclusion

This bibliometric analysis highlights the evolution of research trends in imaging diagnosis of lymphoma, with a focus on advanced modalities like FDG PET-CT and MRI. The study reveals that FDG PET-CT has emerged as a key research hotspot and frontier, demonstrating its pivotal role in modern lymphoma diagnosis. High-impact journals and leading institutions, particularly in the United States and China, dominate the field. These findings provide valuable insights for future research, emphasizing the potential of refining imaging techniques and integrating emerging technologies for improved diagnostic precision and patient outcomes. Over time, research has gradually shifted toward more precise imaging diagnostic tools, such as “PET,” “MRI,” and their “integration.”

## Data Availability

The datasets presented in this article are not readily available because all data generated or analyzed during this study are included in this published article. Requests to access the datasets should be directed to YM, daqiqima@163.com.
