# Supplementary material for: Mapping the landscape and research trend of imaging diagnosis in lymphoma: a bibliometric analysis from 1976 to 2024
Source: Front Med (Lausanne). 2025 Jan 29;12:1516817. doi: 10.3389/fmed.2025.1516817 (PMC11813927; doi:10.3389/fmed.2025.1516817)
Supplement: Supplementary file 1 [file Table_1.docx]

**Table S1.** Bibliometric Indicators of High-Impact Journals.

| **Journal** | **h-index** | **JCR Quartile** | **IF** | **TC** | **TC rank** | **TP** | **TP rank** | **PY start** |
| --- | --- | --- | --- | --- | --- | --- | --- | --- |
| RADIOLOGY | 69 | 1 | 12.1 | 10461 | 2 | 184 | 4 | 1976 |
| JOURNAL OF CLINICAL ONCOLOGY | 65 | 1 | 42.1 | 14610 | 1 | 118 | 11 | 1985 |
| JOURNAL OF NUCLEAR MEDICINE | 61 | 1 | 9.1 | 8345 | 4 | 190 | 3 | 1991 |
| AMERICAN JOURNAL OF ROENTGENOLOGY | 55 | 1 | 4.7 | 7225 | 5 | 192 | 2 | 1978 |
| EUROPEAN JOURNAL OF NUCLEAR MEDICINE AND MOLECULAR IMAGING | 52 | 1 | 8.6 | 4899 | 7 | 180 | 5 | 2002 |
| RADIOGRAPHICS | 45 | 1 | 5.2 | 2417 | 15 | 91 | 16 | 1991 |
| BLOOD | 43 | 1 | 21 | 9947 | 3 | 63 | 29 | 1989 |
| CANCER | 41 | 1 | 6.1 | 2933 | 12 | 88 | 19 | 1978 |
| JOURNAL OF COMPUTER-ASSISTED TOMOGRAPHY | 40 | 4 | 1 | 2378 | 16 | 172 | 7 | 1977 |
| ANNALS OF ONCOLOGY | 39 | 1 | 56.7 | 4684 | 9 | 69 | 27 | 1991 |
| EUROPEAN JOURNAL OF RADIOLOGY | 38 | 1 | 3.2 | 1977 | 19 | 145 | 9 | 1989 |
| EUROPEAN RADIOLOGY | 36 | 1 | 4.7 | 2708 | 14 | 157 | 8 | 1995 |
| AMERICAN JOURNAL OF NEURORADIOLOGY | 33 | 1 | 3.1 | 3351 | 10 | 62 | 30 | 1984 |
| LEUKEMIA & LYMPHOMA | 32 | 3 | 2.2 | 864 | 49 | 195 | 1 | 1993 |
| INTERNATIONAL JOURNAL OF RADIATION ONCOLOGY BIOLOGY PHYSICS | 31 | 1 | 6.4 | 2896 | 13 | 80 | 20 | 1984 |
| NEUROLOGY | 30 | 1 | 7.7 | 1711 | 23 | 39 | 50 | 1981 |
| CLINICAL CANCER RESEARCH | 28 | 1 | 10 | 1492 | 26 | 38 | 51 | 2000 |
| NEURORADIOLOGY | 28 | 2 | 2.4 | 927 | 42 | 75 | 24 | 1980 |
| BRITISH JOURNAL OF HAEMATOLOGY | 27 | 1 | 5.1 | 2284 | 17 | 61 | 31 | 1980 |
| ANNALS OF HEMATOLOGY | 25 | 2 | 3 | 1459 | 27 | 95 | 14 | 1993 |
| Abbreviations: PY start: the year of the journal's starting publication; TP: total publications; TP rank: rank of total publications. TC: total citations; TC rank: rank of total citations. | | | | | | | | |

| **Table S2.** Publication and Citation Profiles of Leading Countries. | | | | | | | | |
| --- | --- | --- | --- | --- | --- | --- | --- | --- |
| **Country** | **Articles** | **Freq** | **MCP ratio** | **TC** | **TC rank** | **TP** | **TP rank** | **Average Article Citations** |
| USA | 2362 | 0.227 | 0.114 | 91816 | 1 | 7984 | 1 | 38.9 |
| CHINA | 1525 | 0.146 | 0.077 | 15537 | 4 | 4749 | 2 | 10.2 |
| JAPAN | 1173 | 0.113 | 0.026 | 17491 | 3 | 3763 | 3 | 14.9 |
| GERMANY | 614 | 0.059 | 0.197 | 18737 | 2 | 2867 | 5 | 30.5 |
| ITALY | 612 | 0.059 | 0.132 | 13174 | 7 | 2880 | 4 | 21.5 |
| KOREA | 441 | 0.042 | 0.057 | 7105 | 9 | 1728 | 7 | 16.1 |
| FRANCE | 398 | 0.038 | 0.224 | 13516 | 5 | 2590 | 6 | 34 |
| UNITED KINGDOM | 354 | 0.034 | 0.15 | 13386 | 6 | 1574 | 8 | 37.8 |
| TURKEY | 263 | 0.025 | 0.042 | 3020 | 15 | 738 | 11 | 11.5 |
| CANADA | 200 | 0.019 | 0.215 | 3821 | 11 | 817 | 10 | 19.1 |
| INDIA | 191 | 0.018 | 0.058 | 2403 | 18 | 563 | 14 | 12.6 |
| NETHERLANDS | 177 | 0.017 | 0.232 | 7671 | 8 | 916 | 9 | 43.3 |
| ISRAEL | 160 | 0.015 | 0.125 | 4751 | 10 | 668 | 13 | 29.7 |
| SPAIN | 134 | 0.013 | 0.179 | 2301 | 19 | 724 | 12 | 17.2 |
| AUSTRALIA | 118 | 0.011 | 0.22 | 2900 | 16 | 550 | 16 | 24.6 |
| SWITZERLAND | 111 | 0.011 | 0.333 | 3681 | 12 | 559 | 15 | 33.2 |
| AUSTRIA | 89 | 0.009 | 0.326 | 2507 | 17 | 386 | 18 | 28.2 |
| BELGIUM | 84 | 0.008 | 0.155 | 3233 | 14 | 393 | 17 | 38.5 |
| DENMARK | 73 | 0.007 | 0.425 | 3250 | 13 | 379 | 19 | 44.5 |
| BRAZIL | 70 | 0.007 | 0.271 | 1201 | 21 | 245 | 21 | 17.2 |
| Abbreviations: Freq: frequency of total publications; MCP ratio: proportion of multiple country publications; TP: total publications; TP rank: rank of total publications. TC: total citations; TC rank: rank of total citations. | | | | | | | | |

| **Table S3.** The top 20 countries in terms of intensity of cooperation between 1976 and 2024. | | | | |
| --- | --- | --- | --- | --- |
| **Rank** | **Countries** | **No. of documents** | **No. of citations** | **Total link strength** |
| 1 | USA | 2801 | 106248 | 1155 |
| 2 | Germany | 781 | 29429 | 727 |
| 3 | UK | 520 | 25624 | 643 |
| 4 | France | 533 | 23795 | 605 |
| 5 | Italy | 749 | 20050 | 537 |
| 6 | Switzerland | 228 | 11439 | 456 |
| 7 | Canada | 316 | 10595 | 370 |
| 8 | Netherlands | 264 | 14962 | 347 |
| 9 | Spain | 211 | 5889 | 336 |
| 10 | China | 1605 | 17890 | 297 |
| 11 | Belgium | 150 | 7467 | 259 |
| 12 | Australia | 179 | 6985 | 233 |
| 13 | Denmark | 117 | 6693 | 219 |
| 14 | Austria | 135 | 3677 | 218 |
| 15 | Sweden | 98 | 4710 | 214 |
| 16 | Japan | 1211 | 19076 | 211 |
| 17 | Czech Republic | 66 | 2018 | 201 |
| 18 | South Korea | 477 | 8965 | 190 |
| 19 | Poland | 77 | 1567 | 176 |
| 20 | Israel | 191 | 6613 | 141 |

| **Table S4.** The top 20 most influential authors between 1976 and 2024, sorted by h-index. | | | | | | | | | |
| --- | --- | --- | --- | --- | --- | --- | --- | --- | --- |
| **Author** | **h-index** | **g-index** | **m-index** | **PY start** | **TP** | **TP rank** | **TP frac** | **TC** | **TC rank** |
| MEIGNAN MICHEL | 21 | 29 | 1.167 | 2007 | 29 | 4 | 2.82 | 3656 | 2 |
| ALBANO DOMENICO | 19 | 28 | 2.111 | 2016 | 38 | 1 | 5.69 | 827 | 18 |
| HUTCHINGS MARTIN | 19 | 26 | 1 | 2006 | 26 | 6 | 4.91 | 2420 | 4 |
| TILLY HERVE | 19 | 27 | 1.056 | 2007 | 27 | 5 | 2.43 | 1689 | 6 |
| HAIOUN CORINNE | 17 | 24 | 0.944 | 2007 | 24 | 10 | 1.55 | 1944 | 5 |
| NIEVELSTEIN RUTGER A. J. | 16 | 28 | 0.941 | 2008 | 36 | 2 | 5.56 | 861 | 15 |
| YAHALOM JOACHIM | 16 | 23 | 0.842 | 2006 | 23 | 11 | 2.37 | 1406 | 9 |
| FISHMAN EK | 15 | 19 | 0.366 | 1984 | 19 | 16 | 5.97 | 946 | 11 |
| KWEE THOMAS C. | 15 | 30 | 0.882 | 2008 | 36 | 2 | 4.97 | 970 | 10 |
| MAYERHOEFER MARIUS E. | 15 | 24 | 1.25 | 2013 | 26 | 6 | 3.08 | 612 | 20 |
| VERA PIERRE | 15 | 20 | 0.833 | 2007 | 20 | 14 | 2.38 | 898 | 14 |
| ZINZANI PL | 15 | 16 | 0.441 | 1991 | 16 | 20 | 1.3 | 843 | 16 |
| ZUCCA EMANUELE | 15 | 25 | 0.882 | 2008 | 25 | 8 | 1.97 | 5195 | 1 |
| CASASNOVAS OLIVIER | 14 | 17 | 0.737 | 2006 | 17 | 19 | 1.26 | 843 | 16 |
| FIJNHEER ROB | 14 | 25 | 0.875 | 2009 | 25 | 8 | 3.32 | 648 | 19 |
| GALLAMINI ANDREA | 14 | 21 | 0.778 | 2007 | 21 | 12 | 2.87 | 901 | 13 |
| KOSTAKOGLU LALE | 14 | 21 | 1 | 2011 | 21 | 12 | 3.43 | 1550 | 7 |
| LUMINARI STEFANO | 14 | 18 | 1 | 2011 | 18 | 18 | 1.12 | 1455 | 8 |
| MOSKOWITZ CRAIG H. | 14 | 19 | 0.933 | 2010 | 19 | 16 | 2.93 | 914 | 12 |
| WAHL RICHARD L. | 14 | 20 | 0.737 | 2006 | 20 | 14 | 3.36 | 3499 | 3 |
| Abbreviations: PY start: the year of the journal starting publication; TP: total publications; TP Freq: articles fractionalized; TP rank: rank of total publications. TC: total citations; TC rank: rank of total citations. | | | | | | | | | |
